# Idiopathic Retroperitoneal Hematoma Associated With Anticoagulation in a Patient With Moderate COVID-19 Pneumonia: A Case Report

**DOI:** 10.7759/cureus.87395

**Published:** 2025-07-06

**Authors:** Hikaru Murooka, Natsumi Yamamoto, Shiho Amano, Chiaki Sano, Ryuichi Ohta

**Affiliations:** 1 Family Medicine, International University of Health and Welfare, Tokyo, JPN; 2 Community Care, Unnan City Hospital, Unnan, JPN; 3 Community Medicine Management, Shimane University Faculty of Medicine, Izumo, JPN

**Keywords:** aged, anticoagulants, covid-19, family medicine, general medicine, heparin, physical examination, retroperitoneal hemorrhage, rural

## Abstract

We report a case of idiopathic retroperitoneal hematoma in an 82-year-old man with moderate COVID-19 pneumonia receiving prophylactic anticoagulation therapy. The patient was initially treated with remdesivir, dexamethasone, and subcutaneous heparin, showing improved respiratory function. On hospital day 6, he developed sudden right lower abdominal pain without prior trauma. Physical examination revealed abdominal distension and tenderness. Laboratory tests showed no evidence of coagulopathy. Contrast-enhanced computed tomography identified active bleeding in the right iliopsoas muscle, consistent with retroperitoneal hematoma. The patient was transferred to a tertiary center for management. This case underscores that retroperitoneal hematoma can occur even in moderately ill COVID-19 patients without abnormal coagulation markers. Vigilant physical examination and early imaging are essential for timely diagnosis. Individualized risk assessment is critical when initiating anticoagulation therapy in elderly patients with comorbidities.

## Introduction

The global pandemic of coronavirus disease 2019 (COVID-19) continues to pose a significant public health challenge and remains an active area of clinical research [1]. COVID-19 pneumonia is now well recognized to substantially increase the risk of thrombotic complications, including deep vein thrombosis (DVT) and pulmonary embolism (PE), particularly among hospitalized patients [2]. Compared to patients admitted for non-COVID-related illnesses, those hospitalized for COVID-19 pneumonia demonstrate a markedly higher incidence of venous thromboembolism (VTE) [3].

Anticoagulation therapy with low-molecular-weight heparin (LMWH) has become a mainstay of treatment for hospitalized COVID-19 patients in Japan, based on evidence demonstrating that such therapy reduces thrombotic events, minimizes organ damage, and improves 28-day mortality outcomes [4]. Recent studies have further indicated that therapeutic-dose anticoagulation, as opposed to prophylactic dosing, significantly improves survival among patients with mild to moderate COVID-19 pneumonia accompanied by hypoxemia, without increasing the incidence of fatal bleeding complications [5].

Despite these benefits, the potential for serious hemorrhagic events during anticoagulation therapy remains a concern [6,7]. Here, we report the case of an elderly male patient with moderate COVID-19 pneumonia who developed a retroperitoneal hematoma while receiving low-dose heparin anticoagulation. Notably, this hemorrhagic complication occurred despite careful monitoring of coagulation parameters, which remained within normal ranges throughout treatment. This case underscores the importance of maintaining vigilance regarding bleeding risks during anticoagulation in COVID-19 patients, even when conventional laboratory markers indicate a stable coagulation status.

## Case presentation

An 82-year-old man initially presented to his primary care physician with fever and sore throat and was diagnosed with mild COVID-19 infection. He subsequently developed COVID-19 pneumonia (moderate disease, stage 2) and was admitted to a rural hospital. Initial treatment included remdesivir 100 mg, dexamethasone sodium phosphate 6.6 mg, and ampicillin-sulbactam (ABPC/SBT) 3 g/day. However, on hospital day 5, the patient’s oxygen requirements increased, and radiologic findings worsened, prompting transfer to our hospital. The patient had received three doses of the COVID-19 vaccine. His history included smoking 20 cigarettes per day until the age of 60 (Brinkman index 800). Comorbidities included hypertension, type 2 diabetes mellitus, chronic renal failure, hyperuricemia, and reflux esophagitis. His regular medications were nifedipine 20 mg, olmesartan 10 mg, febuxostat 10 mg, and lansoprazole 15 mg daily.

Upon admission to the rural hospital, vital signs were as follows: temperature 36.5°C, blood pressure 183/90 mmHg, heart rate 90 bpm, respiratory rate 20 breaths/min, and oxygen saturation (SpO₂) 98% on 3 L/min supplemental oxygen. Physical examination revealed bilateral basal inspiratory crackles and diminished heart sounds without peripheral edema. Laboratory tests demonstrated elevated white blood cell count, lactate dehydrogenase (LDH), Krebs von den Lungen-6 (KL-6), and C-reactive protein (CRP) (Table 1).

**Table 1:** Initial laboratory data of the patient KL-6 = Krebs von den Lungen-6; APTT = activated partial thromboplastin time

Parameter	Level	Reference
White blood cells	13500	3.5-9.1×10^3^/μL
Neutrophils	96.9	44.0-72.0%
Lymphocytes	2.3	18.0-59.0%
Hemoglobin	12.7	11.3-15.2 g/dL
Hematocrit	38.6	33.4-44.9%
Mean corpuscular volume	90.9	79.0-100.0 fL
Platelets	26.8	13.0-36.9×10^4^/μL
Total protein	5.7	6.5-8.3 g/dL
Albumin	2.2	3.8-5.3 g/dL
Aspartate aminotransferase	36	8-38 IU/L
Alanine aminotransferase	26	4-43 IU/L
Lactate dehydrogenase	542	121-245 U/L
Blood urea nitrogen	31.2	8-20 mg/dL
Creatinine	1.62	0.40-1.10 mg/dL
eGFR	32.4	60.0-mL/min/m^2^
Serum Na	135	135-150 mEq/L
Serum K	4.2	3.5-5.3 mEq/L
Serum Cl	99	98-110 mEq/L
Ferritin	746.7	14.4-303.7 ng/mL
C-reactive protein	13.01	<0.30 mg/dL
KL-6	1218	105.3-401.2U/mL
PT (%)	75.1	70-130%
PT-INR	1.16	0.85-1.15
APTT	29.3	25.0-40.0 sec
D-dimer	5.90	-1.00 µg/mL
Urine test	-	-
Leukocyte	Negative	Negative
Protein	Negative	Negative
Blood	Negative	Negative

Arterial blood gas analysis showed a P/F ratio of 196, indicating type 1 respiratory failure with a markedly elevated alveolar-arterial oxygen gradient (A-aDO₂) (Table 2).

**Table 2:** Blood gas result A-aDO₂ and P/F ratio do not have universally established normal ranges but are commonly used to assess gas exchange efficiency. An A-aDO₂ of less than 15-20 mmHg is typically expected in young adults breathing room air, and a P/F ratio of ≥300 generally indicates normal oxygenation. pH = potential of hydrogen; PCO₂ = partial pressure of carbon dioxide; PO₂ = partial pressure of oxygen; HCO₃⁻ = bicarbonate; BE = base excess; tCO₂ = total carbon dioxide; SaO₂ = arterial oxygen saturation; ctHb = total hemoglobin concentration; cNa⁺ = sodium; cK⁺ = potassium; cCl⁻ = chloride; cCa²⁺ = ionized calcium; cGlu = glucose; cLac = lactate; AnGap = anion gap; A-aDO₂ = alveolar-arterial oxygen difference; P/F = PaO₂/FiO₂ ratio

Parameter	Level	Reference
pH	7.513	7.35-7.45
PCO_2_	34.7	35.0-45.0mmHg
PO_2_	58.8	75.0-100.0mmHg
HCO_3_^-^	27.8	20.0-26.0 mmol/L
BE	5.0	-3.0-3.0 mmol/L
tCO_2_	28.9	18.1-24.2 mmol/L
SaO_2_	91.2	92.0-98.5%
ctHb	13.4	13.5-17.6g/dL
cNa^+^	133	136-152mmol/L
cK^+^	4.0	3.5-5.3 mmol/L
cCl-	98	96-107 mmol/L
cCa_2_^+^	1.02	1.13-1.32 mmol/L
cGlu	157	70-105mg/dL
cLac	2.8	0.5-1.6 mmol/L
AnGap	6.5	6.0-15.0 mmol/L
A-aDO_2_	114.2	mmHg
P/F	196	-

**Figure 1:**
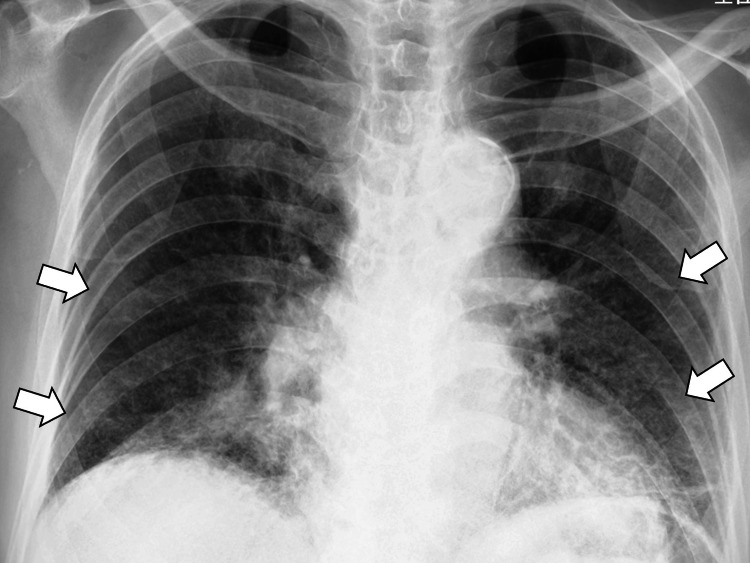
Chest X-ray showing bilateral ground-glass opacities in the lower lung fields (white arrows)

Chest radiograph showed bilateral ground-glass opacities in the lower lung fields (Figure 1).

**Figure 2:**
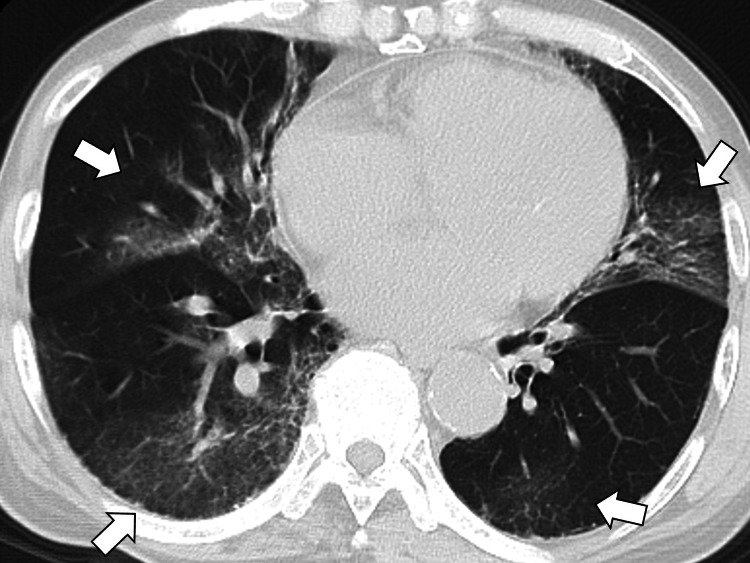
Chest computed tomography showing multiple bilateral subpleural ground-glass opacities (white arrows)

Chest computed tomography (CT) revealed multiple bilateral subpleural ground-glass opacities, which were enlarged compared to prior imaging from the referring hospital (Figure 2).

Echocardiography demonstrated a preserved left ventricular ejection fraction (>50%), no regional wall motion abnormalities, and no valvular regurgitation. The inferior vena cava diameter was 8/2 mm with normal respiratory variability. Based on the progressive respiratory deterioration and imaging findings, the patient was diagnosed with worsening COVID-19 pneumonia and was managed in isolation for five days.

**Figure 3:**
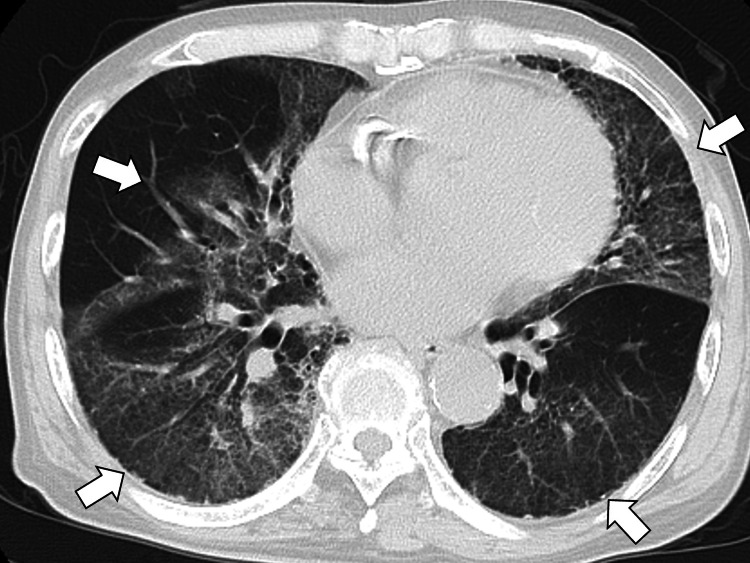
Chest computed tomography showing worsening bilateral infiltrates, suggestive of acute respiratory distress syndrome (white arrows)

Due to the clinical deterioration and worsening CT findings, treatment for acute respiratory distress syndrome (ARDS) was initiated (Figure 3).

Dexamethasone 10 mg was administered to control inflammation, along with tocilizumab 400 mg and baricitinib 2 mg, considering the deteriorating clinical course. Remdesivir 100 mg was continued, and subcutaneous heparin 5000 units was administered twice daily for anticoagulation. Following initiation of this regimen, the patient’s oxygen requirements gradually decreased, and oxygen therapy was tapered. Bedside rehabilitation was initiated on hospital day 2, and the patient regained independence in activities of daily living.

On hospital day 6, the patient developed sudden-onset right lower abdominal pain, peripheral coldness, and decreased oxygen saturation with no changes in the other vital signs. Physical examination revealed right lower abdominal distension and tenderness. Coagulation studies were within normal limits, including activated partial thromboplastin time (APTT) and platelet count (Table 3).

**Table 3:** Changes in coagulation and hematological parameters during heparin therapy APTT = activated partial thromboplastin time; Hb = hemoglobin; Day 0 = baseline (start of heparin therapy)

Parameter	Day 0	Day 3	Day 6
Total heparin dose (units)	5000 units/12hr	10000/day	10000/day
APTT (sec)	29.3	31.6	41.9
Platelet count (×10^4^/µ)	26.8	27.5	20.5
D-dimer (μg/mL)	5.90	-	3.40
Hemoglobin (×10^6^g/dL)	12.7	12.3	12.7

**Figure 4:**
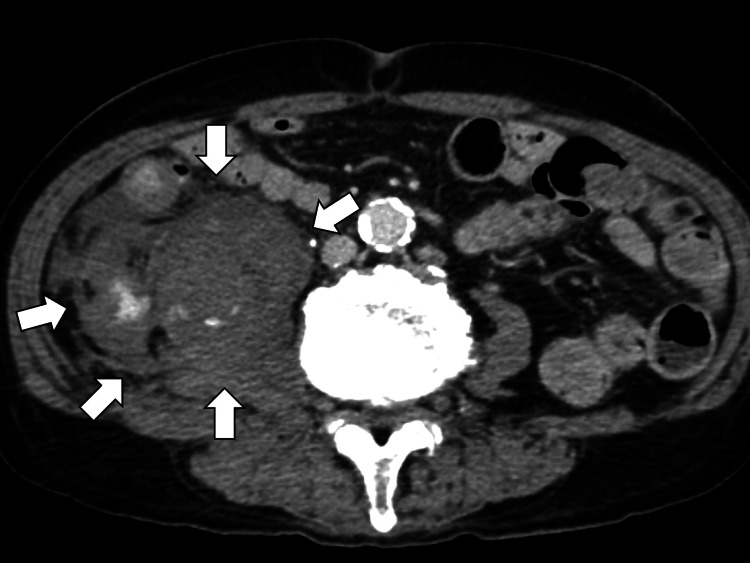
Contrast-enhanced computed tomography showing active extravasation in the right iliopsoas muscle (white arrows)

Contrast-enhanced CT identified active extravasation in the right iliopsoas muscle, consistent with retroperitoneal hemorrhage (Figure 4).

The patient was urgently transferred to a tertiary care center with interventional radiology (IVR) capability for further management.

At the tertiary care center, conservative treatment of the retroperitoneal hematoma was continued, resulting in a gradual decrease in hematoma size. The patient’s overall condition stabilized, and he was scheduled for transfer back to our hospital. However, he subsequently developed pyelonephritis and secondary organizing pneumonia associated with COVID-19 pneumonia. He was treated with nasal high-flow oxygen therapy and methylprednisolone 1000 mg for three days for progressive organizing pneumonia. The methylprednisolone dose was then tapered from 50 mg to 40 mg. Due to prolonged hospitalization, the patient experienced functional decline, and two months later was transferred to the rehabilitation ward of our hospital for continued habilitation.

## Discussion

This case highlights an important but rare complication of anticoagulation therapy, idiopathic retroperitoneal hematoma, in a patient with moderate COVID-19 pneumonia. Despite normal coagulation parameters, the patient developed a significant hemorrhage after receiving prophylactic heparin. Careful physical examination enabled early detection and intervention. The key learning points from this case are (1) idiopathic retroperitoneal hematoma can occur even in patients with moderate COVID-19 and without laboratory signs of coagulopathy; (2) vigilant physical examination and clinical suspicion are crucial for timely diagnosis; and (3) contrast-enhanced CT is an essential tool for confirming hemorrhagic complications in this setting.

Anticoagulation therapy plays a vital role in reducing mortality in patients with COVID-19 pneumonia. Japanese guidelines recommend its use in hospitalized patients with moderate to severe disease [8]. In this case, an elderly patient with moderate COVID-19 pneumonia was treated with standard therapy, including anticoagulation with subcutaneous heparin, yet developed an unexpected retroperitoneal hematoma during the disease course. A Japanese study demonstrated that early initiation of anticoagulation within six hours of ICU admission and maintaining APTT at 1.5-2.5 times baseline significantly reduced mortality [9]. Studies also support prophylactic and therapeutic heparin superiority over no anticoagulation [10]. However, bleeding complications, including retroperitoneal hematomas, are increasingly reported, particularly among critically ill patients [11]. Laboratory signs of coagulopathy often accompany these events. In this case, no abnormal coagulation parameters were observed, despite the occurrence of hemorrhage.

Idiopathic retroperitoneal hematoma is more common in elderly patients, especially those with comorbidities such as chronic renal failure [12]. Our patient had multiple risk factors, including advanced age and renal dysfunction, which likely contributed to hematoma development. Importantly, early diagnosis was made based on physical findings rather than laboratory data alone. Clinicians must consider background risk factors and maintain a broad differential diagnosis [13]. Patient isolation during COVID-19 management limits opportunities for detailed physical examination [14,15]. This can delay recognition of complications such as bleeding or trauma-related events. In our case, vigilant physical examination detected abdominal tenderness early, prompting timely imaging. Regardless of isolation protocols, clinicians must remain attentive to subtle clinical changes when managing anticoagulated COVID-19 patients.

Retroperitoneal hematomas are rarely reported in patients with moderate COVID-19 pneumonia. Further research is needed to clarify bleeding risk across different disease severities and to guide optimal anticoagulation strategies in this population. Retroperitoneal hematoma often presents with nonspecific symptoms, including back or abdominal pain, lumbar plexus disturbance, and hemodynamic instability [16]. In this patient, sudden-onset right-sided abdominal pain was the key clinical clue. APTT monitoring alone may not predict bleeding risk in anticoagulated COVID-19 patients. Physical examination and awareness of symptoms remain crucial for early detection. Imaging is essential for confirmation. CT has a sensitivity of 86.7%-93.3% for detecting retroperitoneal hematoma [16]. While non-contrast CT may lack specificity, contrast-enhanced CT should be performed promptly when bleeding is suspected. Although concerns about contrast use may arise in frail patients, retroperitoneal hematoma carries a high mortality risk, warranting proactive diagnostic efforts [12,16]. Clinicians must balance thromboprophylaxis benefits with bleeding risks, particularly in elderly patients with comorbidities. Early recognition of complications through physical examination, clinical vigilance, timely imaging, and interprofessional collaboration can improve outcomes [17,18].

## Conclusions

We report a rare case of idiopathic retroperitoneal hematoma occurring during anticoagulation therapy in a patient with moderate COVID-19 pneumonia and normal coagulation parameters. This case highlights the importance of maintaining clinical vigilance for hemorrhagic complications, even in patients without laboratory evidence of coagulopathy. Advanced age and comorbidities such as chronic renal failure may increase bleeding risk and warrant careful monitoring. Thorough physical examination played a crucial role in the early detection of this complication, particularly in the context of patient isolation. Prompt use of contrast-enhanced CT enabled timely diagnosis and appropriate management. To improve clinical outcomes, further research is needed to clarify the incidence, risk factors, and optimal anticoagulation strategies in moderately ill COVID-19 patients.
